# Development and Effects of Influenza Antiviral Drugs

**DOI:** 10.3390/molecules26040810

**Published:** 2021-02-04

**Authors:** Hang Yin, Ning Jiang, Wenhao Shi, Xiaojuan Chi, Sairu Liu, Ji-Long Chen, Song Wang

**Affiliations:** Key Laboratory of Fujian-Taiwan Animal Pathogen Biology, College of Animal Sciences (College of Bee Science), Fujian Agriculture and Forestry University, Fuzhou 350002, China; 1190608024@fafu.edu.cn (H.Y.); 1180608008@fafu.edu.cn (N.J.); 1190608016@fafu.edu.cn (W.S.); chixiaojuan88@fafu.edu.cn (X.C.); 1200608006@fafu.edu.cn (S.L.); chenjl@im.ac.cn (J.-L.C.)

**Keywords:** influenza virus, chemosynthetic drugs, plant extracts, microbial metabolites, drug resistance

## Abstract

Influenza virus is a highly contagious zoonotic respiratory disease that causes seasonal outbreaks each year and unpredictable pandemics occasionally with high morbidity and mortality rates, posing a great threat to public health worldwide. Besides the limited effect of vaccines, the problem is exacerbated by the lack of drugs with strong antiviral activity against all flu strains. Currently, there are two classes of antiviral drugs available that are chemosynthetic and approved against influenza A virus for prophylactic and therapeutic treatment, but the appearance of drug-resistant virus strains is a serious issue that strikes at the core of influenza control. There is therefore an urgent need to develop new antiviral drugs. Many reports have shown that the development of novel bioactive plant extracts and microbial extracts has significant advantages in influenza treatment. This paper comprehensively reviews the development and effects of chemosynthetic drugs, plant extracts, and microbial extracts with influenza antiviral activity, hoping to provide some references for novel antiviral drug design and promising alternative candidates for further anti-influenza drug development.

## 1. Background

Influenza virus is a negative-sense, single-stranded RNA virus belonging to the Orthomyxovirus family, influenza virus genus. Influenza viruses can be classified according to their antigenicity into four types—A, B, C and D [1]. Of these, influenza A viruses are the most pathogenic to humans and have a wide range of hosts. Influenza A virus has 18 different hemagglutinin (HA) subtypes (H1–H18) and 11 different neuraminidase (NA) subtypes (N1–N11), which together define the influenza A virus subtype [1,2,3]. The host range of influenza B virus is relatively limited and its pathogenicity to humans is relatively weak. According to epidemiological investigation, no influenza B virus pandemic has ensued so far [3,4,5,6]. Type C influenza virus causes only mild respiratory disease in humans, while type D influenza virus does not appear to be pathogenic to humans [7,8]. The most important characteristic of influenza virus is its variability due to its segmental RNA genome contributing to antigen variation, which makes it extremely difficult to develop vaccines and drugs [9,10,11].

Influenza A virus causes seasonal epidemics worldwide every year and has been responsible for several global outbreaks in history, such as the 1918 Spanish flu and the 2009 H1N1 pandemic [12,13]. Seasonal transmission of influenza virus varies according to geographical location, population size, and population movement in different climatic regions. Understanding the seasonal transmission of influenza virus in different climatic regions can provide theoretical support for optimizing the efficiency of influenza vaccination programs [14]. The annual financial impact of seasonal influenza in China and the world is very large, but not as large as the financial impact of influenza pandemics, such as the worldwide H5N1 and H1N1 outbreaks, which cost countries affected by the influenza virus more than $80 billion in financial losses [15]. Therefore, it can be said that the influenza virus is a serious threat to the safety of public health. Thus far, the World Health Organization still believes that vaccines are the best way to prevent and control an influenza pandemic; however, influenza viruses constantly undergo genetic changes and require vaccines that match the circulating influenza strains to be effective, so seasonal influenza vaccines have to be modified annually [16,17,18,19]. In addition, the application of vaccines has also been limited due to the side effects and storage difficulty [17]. Therefore, the study of antiviral drugs is increasingly imperative [16,20]. In this paper, the development and effects of anti-influenza drugs from different sources are reviewed in order to provide new ideas for the prevention and control of influenza in future.

## 2. Influenza Virus Invades Host Mechanisms

When influenza virus invades host cells, the HA protein of the virus first binds to cellular receptors with α-2,3-linked or α-2,6-linked sialic acid. After that, influenza virus is internalized through a variety of endocytic pathways, including cypermethrin-dependent and non-cypermethrin-dependent pathways [21,22,23]. The cation channel of the M2 ion channel protein of the virus then opens, reducing the pH value inside the virus, allowing the viral envelope to fuse with the endosomal membrane in a low pH-dependent manner, and the viral genome is released into the cytoplasm, where it is further transported to the nucleus to begin genome replication [24,25]. During this process, influenza virus HA, NA, M2, and vRNP complex play critical roles, making them potential targets for the development of anti-influenza drugs. HA is a membrane protein that exists on the surface of the virus and is composed of HA1 and HA2 [26,27,28]. Highly pathogenic avian influenza virus strains (H5 and H7 subtypes) have the ability to infect humans due to their HA receptor binding site (RBS) mutation, thereby enhancing the affinity of the virus to the cell surface receptor (α-2,6-linked sialic acid) [27,29]. In addition to binding to host cells, the second major function of HA is to mediate viral and cell membrane fusion [30,31]. This fusion process is essential for the introduction of the viral genome into cells [32,33,34]. Indeed, new antiviral drugs have been developed using HA as an antiviral target [35].

As an important weapon for influenza virus to destroy host cell receptor, NA can prevent the accumulation of virus particles on the surface of host cells caused by the adsorption function of HA, and can promote the release of virus progeny particles, which also play an important role in the process of influenza virus infection of host cells [36,37]. HA-mediated attachment and NA-mediated release of influenza viruses need to keep a balance in order to allow productive influenza virus infection [38,39,40]. NA inhibitors are extensively used in the treatment and prophylaxis of influenza virus infection presently. 

**Figure 1:**
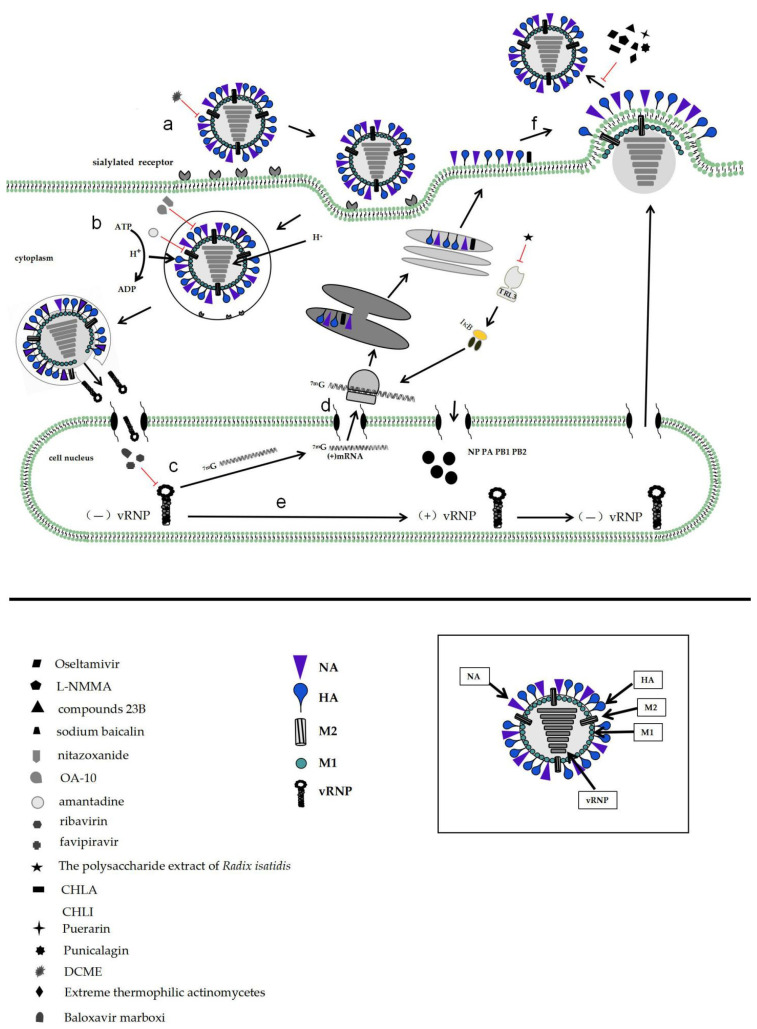
Replication cycle of influenza virus and targets of anti-influenza drugs. (a) Influenza virus hemagglutinin (HA) binds to sialylated host cell receptors, and then is internalised into endosomes through multiple endocytosis pathways. (b) Acidification of the endosome leads to activation of the M2 proton channel and virion acidification, resulting in virus uncoating and the release of viral genome into the cytoplasm, where it is further transported to the nucleus to begin genome replication. (c) In the nucleus, influenza virus begins to synthesize viral mRNAs. (d) HA, neuraminidase (NA) and M2 are processed in the Golgi body and the endoplasmic reticulum, and then transported to the cell surface. (e) Influenza virus polymerase can synthesize both viral mRNAs and vRNAs. vRNAs are first converted into positive-stranded cRNAs, and then new vRNAs can be synthesized using cRNAs as templates. (f) Viral proteins and genomic RNA are transported to the cell surface to assemble progeny viruses. Then, influenza virus neuraminidase (NA) cuts off the HA-receptor bond to allow progeny viruses to be released from the surface of the infected cell and proceed to infect new cells. The sites of action of antiviral drugs are shown in red.

The vRNP complex consists of eight negative-sense, single-stranded RNAs, nuclear protein, and RNA polymerases, which are the basic units of influenza virus replication [41]. The viral proteins that make up vRNP all have nuclear localization sequences (NLS). vRNP is assembled in the cytoplasm and then enters the nucleus through nuclear localization to complete viral replication and transcription [41,42]. The influenza virus polymerase plays a major role in the replication and transcription of influenza virus. Polymerase synthesizes viral mRNA via a unique “cap snapping” mechanism using short-end primers from cellular transcripts [43]. Interference with the activity of the RNA-dependent RNA polymerase (RdRP) is an effective means to reduce viral resistance and inhibit viral replication, and viral RdRP is one of the most promising targets for the development of novel influenza antiviral drugs [43,44,45,46,47]. As a non-structural protein of the influenza virus, NS1 protein can regulate the viral life cycle, the immune function of the host, and play an auxiliary role in the process of influenza virus infection of host cells [48]. It has been reported that compounds A9 and A22 inhibit the replication of influenza virus and the function of NS1 by blocking the interaction between CPSF30 and the NS1 effector domain, and the NS1 protein is also expected to be an important target for the development of new influenza antiviral drugs [48,49,50]. The replication cycle of influenza virus and targets of anti-influenza drugs are depicted in Figure 1.

## 3. Development and Effects of Influenza Antiviral Drugs

Influenza viruses pose a significant threat to public health, strongly associated with their high variability and recombination [51,52,53]. The known influenza virus strains constantly mutate and the genomic segments may undergo reassortment to form new virus subtypes [54,55]. Because of the variable nature of the viruses themselves, the development of vaccines and drugs is facing great challenges. Nevertheless, research has led to the development of two main types of compound drugs in clinical treatment of influenza virus—NA inhibitors and M2 channel ion blockers [56,57]. NA inhibitors can inhibit the NA activity of influenza viruses, weaken the release of influenza virus particles from infected cells, and thus effectively inhibit the replication of the viruses. Among them, the most representative NA inhibitors are oseltamivir, peramivir, and zanamivir [58,59]. M2 channel ion blockers mainly inhibit viral replication by blocking the hydrogen ion channel activity of M2 protein of influenza virus. Representative drugs are amantadine and rimantadine [60].

Because of resistance problems faced with the influenza virus for NA inhibitors and M2 channel blocker drugs, research has focused on the influenza virus RNA polymerase as a drug target due to its important role in regulating influenza virus replication and transcription and the highly conserved RNA polymerases between different strains [61]. There are multiple potential antiviral drugs that could lead to effective antiviral activity, including ribavirin and favipiravir [62]. In addition, because of the overuse of compound drugs, a large number of drug-resistant strains have emerged. Natural antiviral drugs are also being explored; in clinical application, traditional Chinese medicine has shown ideal antiviral activity for drug-resistant strains, without the development of drug resistance problems. These medicines include honeysuckle, Radix isatidis, Terminalia chebula, puerarin, and Yinqiao powder, among others.

### 3.1. Development and Effects of Chemical Synthesis Drugs on Influenza Virus Resistance

Oseltamivir has been widely used in the treatment of influenza virus. Oseltamivir can inhibit the replication of influenza virus by binding to the NA active site as competitive inhibitors [63,64]. However, due to the evolution of influenza virus and the abuse of influenza antiviral drugs, a large number of drug-resistant strains have emerged, for example, the H274Y/H1N1 influenza virus, which is the culprit leading to the H1N1 pandemic, causing huge economic losses [64,65,66]. Therefore, the development of new drugs is urgent. In addition, adverse effects after the clinical use of oseltamivir, such as inhibiting the production of viral antigens, leading to the reduction of acquired antiviral humoral immunity and increasing the probability of re-infection [67], have been observed. In order to reduce the impact of adverse effects, Takahashi et al. demonstrated that *Lactobacillus bulgaricus* OLL1073R-1YC has the ability to stimulate host humoral immunity against influenza virus and can assist oseltamivir in the treatment of influenza virus [67]. L-NMMA, nitazoxanide, etc., have emerged to synergistically fight against influenza viruses in order to make the use of drugs more efficient [68]. L-NMMA is a nitric oxide inhibitor that can be used in collaboration with oseltamivir in the treatment of the H1N1 influenza virus. Smee et al. demonstrated, through animal experiments, that the synergistic effect of the two drugs can significantly reduce the mortality [69]. Nitazoxanide is a thiazole compound that works in conjunction with NA inhibitors against influenza viruses. Different from other influenza antiviral drugs, it does not inhibit the expression of viral proteins but inhibits the replication of influenza virus by blocking HA terminal glycosylation and intracellular transport [70]. An ongoing clinical trial has shown the significant antiviral activity of Nitazoxanide against a wide range of human and avian IAVs as well as various non-influenza respiratory viruses, indicating a wide and bright application foreground for the treatment of respiratory infections [70,71]. Some drugs have also been developed to target current strains resistant to oseltamivir, such as pyridine-containing oseltamivir derivative compounds 23B and sodium baicalin. Compound 23B had a significant inhibitory effect on H5N1 NA as well as a strong inhibitory effect on oseltamivir-resistant A/Liaoning Zhenxin/1109/2010 (H1N1) virus [72]. Like oseltamivir, sodium baicalin is also an inhibitor of viral NA. Jin et al. confirmed that sodium baicalin has an obvious inhibitory ability against the H1N1-H275Y virus strain that is oseltamivir-resistant. However, the use of sodium baicalin in clinical practice is hindered because of its poor water-solubility [73].

The most critical step for influenza virus to infect the host is the interaction between the viral membrane protein HA and the host cell surface receptor mediating the entry of the virus, and certain drugs can interact with the HA protein to alter the biological structure and function of HA, thus preventing the virus from infecting the host [74,75]. As a derivative of oleanolic acid that possesses notable antiviral activity, OA-10 also has a significant inhibitory effect on influenza virus, including H5N1, PR8 (H1N1), H9N2, and H3N2 [76]. It acts by blocking the conformational changes of the HA2 subunit required for viral–endosomal membrane fusion, which is necessary for the release of viral genome from its protective capsid to enable the nucleic acid to be transported into the nucleus, thereby inhibiting the replication of influenza virus [76].

When M2 channel blockers are widely used in clinical practice, they also face the same disadvantages as NA inhibitors such as oseltamivir, producing drug-resistant strains such as the S31N influenza strains that are prevalent in influenza viruses [77]. The replication process of influenza virus requires the activity of the M2 ion channel; amantadine has the ability to inhibit the replication of influenza virus by inhibiting the activity of the M2 protein [78,79,80]. However, due to the mutation of the 31st amino acid in the M2 protein, amantadine loses its ability to inhibit the virus and cannot be used in the clinic [79,80,81]. Therefore, addressing drug resistance is an important issue in the research and development of new drugs.

The emergence of RNA polymerase inhibitors, such as ribavirin and favipiravir, has an exciting impact on the spread and drug resistance of influenza viruses [82]. Ribavirin, a nucleoside compound, has a wide antiviral activity range, and has a good inhibitory ability against A/Vietnam/1203/04 (H5N1) virus and A/Turkey/15/06 (H5N1) virus [83]. Its mechanism involves a reduction in the content of GTP in cells by competitively inhibiting IMP dehydrogenase and a reduction in the replication ability of influenza virus by inhibiting the function of influenza RNA polymerase [83]. Of note, the drug is particularly effective against the H5N1 influenza virus and its combination with oseltamivir is more effective than the single drug [83]. However, the anti-influenza virus effect of ribavirin was only performed in a mouse model, and its effect in human clinical trials was less clear [84]. As an emerging antiviral drug against influenza virus, favipiravir has good antiviral ability against the whole RNA virus, and has been licensed as an anti-influenza drug in Japan [85,86]. It acts by inhibiting the activity of influenza virus RNA polymerase and reducing its conservatism, so that the virus gene mutates [85]. There have been no reports of drug resistance to favipiravir. There are two hypotheses for this—first, it can increase the deleterious mutation rate of the entire genome of the virus and lead to virus extinction; second, the powerful antiviral ability of favipiravir enables the influenza virus to be destroyed before it mutates [87]. Ormond et al. demonstrated that the combination of favipiravir and oseltamivir could explore potential genes for resistance to oseltamivir strains [88]. Because of its nature, favipiravir is likely to become a core drug during the next pandemic and stockpiling of novel drugs is now an important strategy for dealing with future influenza pandemics [85]. In addition, Baloxavir marboxil, which has been approved for the treatment of uncomplicated influenza in otherwise healthy and high-risk patients in numerous countries, can potently inhibit influenza virus production by selectively blocking the catalytic center of polymerase acid (PA) protein in the RNA polymerase complex. However, amino acid substitutions such as I38N/R, E23K/G, A37T, and E199G in the PA subunit bring about new challenges for effectiveness of the drug [89,90,91,92,93].

**Figure 2:**
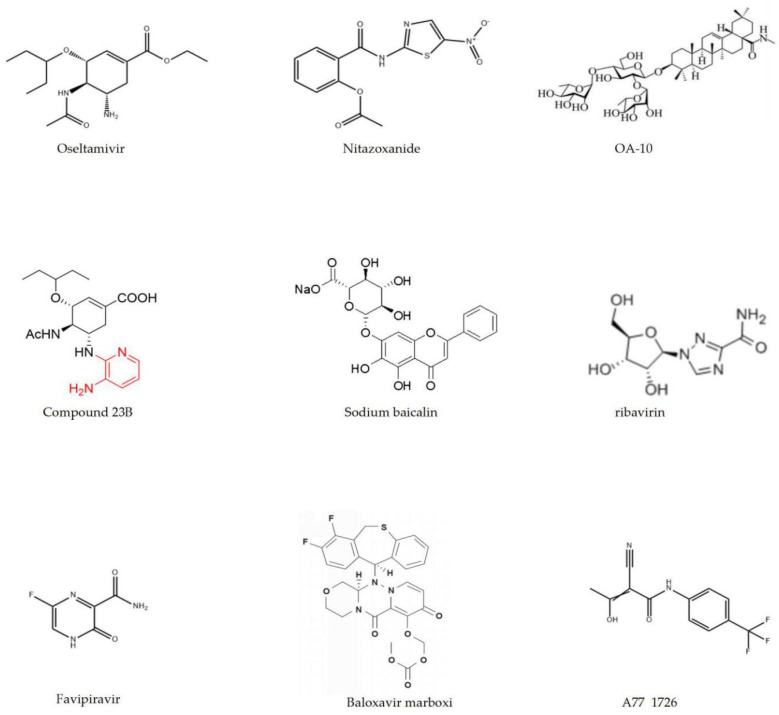
The molecular structures of chemical synthesis drugs.

There are also drugs that have multiple functions, such as leflunomide. As a well-known anti-inflammatory drug primarily used for treating rheumatoid arthritis, leflunomide also shows influenza antiviral activity [94]. Wang et al. found that leflunomide’s metabolite A77 1726 has an inhibitory effect on JAK2 activity. JAK2 not only regulates the function of immune cells, but also plays a significant role in the process of influenza virus replication, indicating that the inhibition of JAK2 activity by drugs is directly related to their antiviral activity [94]. The details of chemical synthesis drugs with anti-influenza activity are listed in Table 1, and their molecular structures are shown in Figure 2.

### 3.2. Development and Effects of Plant Extracts on Influenza Virus Resistance

Due to the limitations in the development of compound drugs, the combination of natural drugs of medicinal plants with empirical knowledge provides a new platform for the development of new antivirals [95]. Honeysuckle, *Radix isatidis*, *T. chebula* and puerarin, as representatives of natural drugs, have had a long history in treating influenza virus in China [96] (Table 2). Studies have shown that honeysuckle has many antiviral active extracts, such as acids extract, flavonoids extract, honeysuckle acids-flavonoids mixture, etc. [97]. In particular, honeysuckle acids-flavonoids mixture showed the strongest antiviral activity against H1N1, H3N2 and the oseltamivir-resistant strain H1N1-H275Y, the flavonoids extract exerted the strongest inhibitory effect on H7N9 influenza virus in vitro, while honeysuckle acids extract was demonstrated to exhibit the most potent therapeutic efficacy against H1N1 influenza virus infection in vivo [97]. The mechanism underlying the inhibitory effects of the extracts on influenza viruses is similar to that of oseltamivir [97].

The polysaccharide extract of *Radix isatidis* has strong influenza antiviral activity and ideal effects on the inhibition of H1N1 and H9N2 influenza viruses [98]. Isatidis polysaccharide has a strong inhibitory effect on the expression of host TLR3 protein, which further reduces the expression of virus-induced pro-inflammatory cytokines and the inflammatory response [99]. As a virus pattern recognition receptor, TLR3 plays a major role in the process of virus infection. It can not only stimulate the production of interferons and some antiviral substances but also induce the spread of the virus, leading to deterioration of the disease [98,99].

In addition, *T. chebula*, as a common Chinese medicine, has a strong influenza antiviral activity. Li et al. determined that chebulagic acid and chebulinic acid in *T. chebula Retz* had strong influenza antiviral activity with IC_50_ values of 1.36 ± 0.36 µM and 1.86 ± 0.98 µM, respectively [100]. It acts by inhibiting the activity of virus NA protein and blocking the release of virus progeny particles to inhibit virus replication [100]. Puerarin is a flavonoid extracted from *Pueraria lobata*, which has many functions, including a significant effect against influenza virus, especially against H1N1 influenza virus [101]. According to animal experiments using a mouse model, Puerarin exhibited effective antiviral activity and had shown no significant side effects after two months of treatment [101]. It acts by inhibiting the NA activity of influenza virus and blocking the nuclear output of the nuclear protein [101]. Some plants that are not included in traditional Chinese medicine, such as pomegranate and ginger, can also exert antiviral activity. Punicalagin, a broad-spectrum influenza inhibitor derived from pomegranate, has an inhibitory effect on different subtypes of influenza virus by inhibiting the NA protein activity of influenza virus and then blocking the release of progeny virus [102]. It is worth noting that Punicalagin also had a significant inhibitory effect on oseltamivir-resistant strains [102]. In addition, the ginger extract Gingerenone A acts as a dual inhibitor of JAK2 and p70S6 kinase (S6K1) to inhibit influenza virus replication by inhibiting JAK2 activity and interfering with viral assembly [103].

As a mixture of various plant extracts, Chinese herbal formulae, such as Gegen Qinlian soup, Lianhua-qingwen capsule, and Yinqiao powder, have been shown to have potent antiviral activity in the clinic [104,105,106] (Table 3).

It has been reported that Gegen Qinlian decoction can downregulate the expression of some signaling pathway factors and the activity of NF-κB, inhibiting the expression of inflammatory factors and the cytokine storm, affecting the differentiation of CD4^+^ T cells and thereby increasing the antiviral immunity of the host [107,108]. The Gegen Qinlian decoction activates the host’s homeostatic inflammatory response, limits immunopathological damage, and improves clinical symptoms [107,108]. Similarly, Lianhua-qingwen is a traditional Chinese medicine prescription for the treatment of respiratory diseases that can relieve clinical symptoms such as fever, cough, sore throat, and fatigue and has a broad-spectrum inhibitory effect on influenza virus [109]. Lianhua-qingwen acts by inhibiting the nuclear output of virus RNP by inhibiting the activity of NF-κB as well as by regulating the immune response after virus infection [109]. Yinqiao powder also has a good inhibitory effect on H1N1 influenza virus and can relieve respiratory symptoms such as cough, headache, fever and so on. It can be used in combination with the Xijiao Dihuang decoction to enhance its antiviral activity [110]. Yinqiao powder exerts influenza antiviral activity by regulating the TLR7/NF-κB signaling pathway [110]. It can also modulate the dysregulated miRNA and mRNA involved in the ERK/JNK-AP-1, IFN-β/STAT signaling pathways, thus improving the host’s antiviral immunity and reducing the damage induced by the inflammatory response [111].

### 3.3. Development and Role of Microbial Metabolites in Influenza Virus Resistance

Besides the plant extracts with good influenza antiviral activity, a large number of effective active antiviral substances have been found in microbial metabolites (Table 4).

The metabolites of actinomycetes have always played an important role in the discovery of new drugs, and most antibiotic drugs are derived from them [112]. Recently, it has been found that the metabolites of actinomycetes also have good antiviral ability [112]. For example, Streptomyces sp (SMU-03) found in Yunnan can produce an antiviral substance, dichloromethane extract (DCME), with a very good inhibitory effect on H1N1 and H3N2 influenza viruses [113]. DCME can block the binding of viral HA protein to the cell surface receptors, thus preventing the virus from entering host cells to produce an antiviral effect [113]. In addition, an extract of extreme thermophilic actinomycetes was found to have a broad-spectrum effect against influenza virus in the harsh environment of Kazakhstan [114]. Berezin et al. proved that the extracts of extremophilic actinomycetes strains K-192-2S, K-340-2S, and K-362-2N had significant antiviral activity through the inhibition of NA activity of all tested strains of influenza A virus, indicating that their commercial value may be higher than that of oseltamivir [114].

In recent years, metabolites of marine organisms have also attracted extensive attention owing to their greater diversity in structure and function compared to terrestrial organisms [115]. Actinomycetes are widely distributed in the ocean and can produce metabolites with diverse biological activities. For example, the sea Verrucosispora is a rare actinomycetes that can secrete a variety of biologically active metabolites, such as Compounds 1–3, with a good inhibitory effect on H1N1 influenza virus [115]. In addition, Wang et al. isolated a new sesterterpene called L435-3 from the phytopathogenic fungus *Bipolaris oryzae* and proved that L435-3 has strong antiviral activity against influenza, including against WSN and PR8 viruses [116,117]. L435-3 acts by inhibiting the replication of influenza virus through increasing the production of type III interferon and some interferon-stimulated genes (ISGs) in the host, thus enhancing the antiviral ability of the host [116].

## 4. Conclusions

Although the development of influenza antiviral drugs has greatly reduced the mortality rate, influenza virus still poses a great threat to public health worldwide due to the emergence of synthetic drug-resistant strains. Therefore, new drugs that do not induce resistance and with unique pharmacological activity need to be discovered and developed. Natural antiviral drugs have many advantages over synthetic drugs, mainly manifested in a lower resistance and mild adverse clinical reactions. Further research and development and more appropriate management of traditional Chinese medicines are required such that these products can be accepted by medical systems worldwide. Meanwhile, more attention should be paid to microbial metabolites for the discovery of new antiviral drugs with high efficiency and safety. Importantly, a better understanding of the mechanisms by which plant extracts and microbial metabolites achieve the anti-influenza action will be beneficial to the development and improvement of antiviral drugs. We have herein summarized the development and effects of three kinds of anti-influenza virus drugs, hoping to provide new ideas for future drug design and development of innovative drugs.

## Figures and Tables

**Table 1:** List of chemical synthesis drugs.

Compounds	Source	Function	IC_50_/EC_50_
Oseltamivir [64]	Shikimic acid	A/Brisbane/59/2007 (H1N1)	IC_50_ = 49.8 ± 6.8 nM
Nitazoxanide [70]	—	A/Puerto Rico/8/1934 (H1N1)	IC_50_ = 3.2 ± 0.0 μM
		A/WSN/1933 (H1N1)	IC_50_ = 1.6 ± 0.2 μM
		A/California/7/2009 (H1N1)	IC_50_ = 3.2 ± 0.0 μM
		A/Parma/24/2009 (H1N1)	IC_50_ = 1.9 ± 0.0 μM
		A/Parma/06/2007 (H3N2)	IC_50_ = 1.0 ± 0.0 μM
		A/Chicken/Italy/9097/1997 (H5N9)	IC_50_ = 3.2 ± 0.5 μM
		A/Goose/Italy/296,246/2003 (H1N1)	IC_50_ = 3.2 ± 0.2 μM
		A/Turkey/Italy/RA5563/1999 (H7N1)	IC_50_ = 1.6 ± 0.2 μM
Compound 23B [72]	—	A/LiaoNing-ZhenXing/1109/2010 (H1N1)	EC_50_ = 14.31 ± 2.59 µM
		A/Puerto Rico/8/1934 (H1N1)	EC_50_ = 12.68 ± 8.96 µM
Sodium baicalin [73]	baicalin	A/FM/1/47 (H1N1-H275Y)	EC_50_ = 20.1 ± 2.3 µM
		A/FM/1/47 (H1N1)	EC_50_ = 4.0 ± 1.1 µM
		A/Beijing/32/92 (H3N2)	EC_50_ = 2.7 ± 1.2 µM
OA-10 [76]	Oleanolic Acid	H5N1	EC_50_ = 14.0 ± 2.3 μM
		PR8 (H1N1)	EC_50_ = 6.7 ± 1.4 μM
		H9N2	EC_50_ = 15.3 ± 2.5 μM
		H3N2	EC_50_ = 19.6 ± 3.7 μM
Ribavirin [82]	—	influenza virus A/X-31 strain	EC_50_ = 8.1 ± 1.3 µM
Favipiravir [85]	—	all influenza virus tested	EC_50_ = 0.014~0.55 µg/mL
Baloxavir marboxi [93]	—	influenza A virus	IC_50_ = 1.4~3.1 nM
		influenza B virus	IC_50_ = 4.5~8.9 nM
A77 1726 [94]	leflunomide	H1N1, H5N1, H9N2	IC_50_^a^ < 50 µM

^a^ A77 1726 inhibits the activity of JAK2 with this IC_50_ value.

**Table 2:** List of plant extracts.

Original Plant	Active Fraction	Function	IC_50_/EC_50_
Honeysuckle [97]	Acids extract	H1N1	IC_50_ = 112.3 ± 17.7 μg/mL
		H3N2	IC_50_ = 332.6 ± 34.5 μg/mL
		H7N9	IC_50_ = 55.9 ± 5.1 μg/mL
		H1N1-H275Y	IC_50_ = 150.4 ± 13.6 μg/mL
	flavonoids extract	H1N1	IC_50_ = 90.9 ± 8.6 μg/mL
		H3N2	IC_50_ = 196.0 ± 23.4 μg/mL
		H7N9	IC_50_ = 24.7 ± 2.3 μg/mL
		H1N1-H275Y	IC_50_ = 108.4 ± 17.0 μg/mL
	acids-flavonoids mixture	H1N1	IC_50_ = 100.1 ± 11.4 μg/mL
		H3N2	IC_50_ = 203.8 ± 9.9 μg/mL
		H7N9	IC_50_ = 35.2 ± 3.1 μg/mL
		H1N1-H275Y	IC_50_ = 125.7 ± 14.7 μg/mL
Radix isatidis [98]	Polysaccharide	A/Chicken/Guangdong/1996 (H9N2)	IC_50_ = 20.57 ± 0.25 mg/mL
		A/PR/8/34 (H1N1)	IC_50_ = 20.48 ± 0.31 mg/mL
		A/Guangzhou/GIRD07/09 (H1N1)	IC_50_ = 8.47 ± 0.07 mg/mL
		A/Aichi/2/68 (H3N2)	IC_50_ = 4.35 ± 0.05 mg/mL
		A/Duck/Guangdong (H6N2)	IC_50_ = 28.20 ± 0.49 mg/mL
Terminalia chebula Retz [100]	CHLA	reporter virus PR8-PB2-Gluc	IC_50_ = 1.36 ± 0.36 µM
	CHLI	reporter virus PR8-PB2-Gluc	IC_50_ = 1.86 ± 0.98 µM
Pueraria lobata [101]	Puerarin	A/FM/1/1947 (H1N1)	EC_50_ = 52.06 μM
Pomegranate [102]	Punicalagin	PR8-PB2-Gluc (H1N1)	IC_50_ = 1.25 ± 0.06 μM
Ginger [103]	Gingerenone A	H5N1	IC_50_ = 10.2~24.5 µM
		H9N2	IC_50_ = 12 µM
		H1N1	IC_50_ = 10.2 µM

**Table 3:** List of Traditional Chinese Medicine mixtures.

Prescriptions	Relieve Symptoms	Mechanism	Composition
GQD [107]	Cough, fever, anti-inflammatory	Down-regulating the expression of TLR signaling pathway factors, and affecting the differentiation of CD4^+^ T cells, thus limiting immune pathological injury caused by virus infection	*Radix puerariae*, *Radix scutellariae*, *Rhizoma coptidis*, *Radix glycyrrhizae*
LH-C [109]	Fever, cough, sore throat, fatigue	Inhibiting the activity of NF-κB and blocking the nuclear export of the viral RNP	*Forsythia suspensa*, *Ephedra sinica Stapf*, *Lonicera japonica Thunb*, *Isatis indigotica Fortune*, *Mentha haplocalyx Briq*, *Dryopteris crassirhizoma Nakai*, *Rhodiola rosea* L ., *Gypsum Fibrosum* , *Pogostemon cablin (Blanco) Benth*, *Rheum palmatum* L ., *Houttuynia cordata Thunb*, *Glycyrrhiza uralensis Fisch*, *Armeniaca*
Yinqiao Powder [110]	Cough, headache, fever	Playing an important anti-influenza role by regulating the TLR7/NF-κB signaling pathway	*honeysuckle*, *Forsythiae fructus*, *Balloon flower root*, *Mint*, *Licorice root*, *Herba lophatheri*, *Fermented soybean*, *Schizonepeta spike*, *Great Burdock Achene*

**Table 4:** List of microbial extracts.

Microbial	Active Fraction	Function	IC_50_/EC_50_
SMU-03 [113]	DCME	A/PR/8/34 (H1N1)	IC_50_ = 0.37 ± 0.22 μg/mL
		A/FM/1/47 (H1N1)	IC_50_ = 0.81 ± 0.09 μg/mL
		A/Aichi/2/68 (H3N2)	IC_50_ = 14.44 ± 0.79 μg/mL
		influenza B virus	IC_50_ = 0.66 ± 0.03 μg/mL
Extreme thermophilic Actinomycetes [114]	K-192-2S	H1N1, H3N2, H5N3, H7N1	EC_50_ = 0.80~0.14 mg/mL
	K-340-2S	H1N1, H3N2, H5N3, H7N1	EC_50_ = 0.05~0.15 mg/mL
	K-362-2N	H1N1, H3N2, H5N3, H7N1	EC_50_ = 0.05~0.20 mg/mL
MS100137 [115]	Compound 1	H1N1	EC_50_ < 10 μM
	Compound 2	H1N1	EC_50_ < 10 μM
	Compound 3	H1N1	EC_50_ < 10 μM
Bipolaris oryzae [116]	L435-3	A/WSN/1933 (H1N1)	IC_50_ = 0.365 μM
		A/PR/8/34 (H1N1)	IC_50_ = 0.391 μM

## Data Availability

Data sharing not applicable.
